# The subfamily Thorictinae (Coleoptera, Dermestidae) from Saudi Arabia

**DOI:** 10.3897/zookeys.1029.63940

**Published:** 2021-04-08

**Authors:** Jiří Háva, Mahmoud S. Abdel-Dayem, Hathal M. Aldhafer

**Affiliations:** 1 Forestry and Game Management Research Institute, Strnady 136, CZ-252 02 Praha 5 – Zbraslav, Czech Republic Forestry and Game Management Research Institute Zbraslav Czech Republic; 2 King Saud University Museum of Arthropods (KSMA), Plant Protection Department, College of Food and Agricultral Sciences, King Saud University, P.O. Box 2460 Riyadh 11451, Saudi Arabia King Saud University Riyadh Saudi Arabia

**Keywords:** Beetles, new species, Saudi Arabia, taxonomy, Thaumaphrastini, Thorictini, *
Thorictus
*, *
Thorictodes
*

## Abstract

In this study, the Saudi Arabian Thorictinae beetle species, *Thorictus riyadhensis* Háva & Abdel-Dayem, **sp. nov.**, *T. shadensis* Háva & Abdel-Dayem, **sp. nov.**, *T. sharafi* Háva & Abdel-Dayem, **sp. nov.**, *T. hanifahensis* Háva & Abdel-Dayem, **sp. nov.** are described, illustrated, and compared with related species. Three other species: *T. castaneus* Germar, 1834; *T. foreli* Wasmann, 1894; and *T. peyerimhoffi* Chobaut, 1904 are excluded from the fauna of Saudi Arabia. A list of Thorictinae species from the Arabian Peninsula is provided.

## Introduction

Thorictinae Agassiz, 1846, with 189 described species worldwide, is a myrmecophilous subfamily of the family Dermestidae (Coleoptera) (Háva 2020a, 2021). Its members can be recognized by their small size, strongly convex and strongly hardened bodies, reduced or absent eyes, absent wings, and their rounded hind coxae that do not reach the outer edge of the metasternum (Háva 2004; Leschen et al. 2010). Thorictinae are currently divided into two tribes, Thaumaphrastini and Thorictini, and include four genera: *Thorictodes* Reitter, 1875, *Afrothorictus* Andreae, 1967, *Macrothorictus* Andreae, 1967, and *Thorictus* Germar, 1834 (Háva 2020a). The genus *Thorictodes* comprises only five species (Herrmann et al. 2011). Two species in *Afrothorictus* and seven species in *Macrothorictus* are known (Háva 2020a). The genus *Thorictus* currently includes 175 species and subspecies from the Palearctic, Oriental, and Afrotropical Regions (Háva 2015a, 2020a).

Thorictinae fauna, in the Arabian Peninsula in general and in Saudi Arabia in particular, is poorly studied due to the lack of adequate dedicated investigation and scant published records for this group. So far, only four species are known from the Arabian Peninsula (Háva 2010, 2021; Abdel-Dayem et al. 2017). *Thorictus peyerimhoffi* Chobaut, 1904 was the first species to be described from Saudi Arabia (Háva 2010) and is now excluded from the Saudi fauna due to misplacement of the type locality “Kasr-er-Rabbat”, to Saudi Arabia. The second species, *T. arabicus*, was described by Háva (2010) in the Eastern Province of Saudi Arabia. In their work on the beetle fauna of Rawdhat Khorim National Park, Central Saudi Arabia, Abdel-Dayem et al. (2017) reported *Thorictodes heydeni* Reitter, 1875, *T. castaneus* Germar, 1834, and *T. foreli* Wasmann 1894. However, *T. castaneus* and *T. foreli* are now excluded from the Saudi fauna due to misidentifications. Recently, Háva (2021) described *Thorictus omanensis* from Oman.

While examining myrmecophilous dermestid specimens from Saudi Arabia, four new species of *Thorictus* were determined and are described below. The present study follows the revision of Thorictinae from the Afrotropical Region (Háva 2013, 2014, 2015b, 2020b).

## Material and methods

### Measurements

The size of beetles’ bodies or body parts can be useful in species recognition; thus, the following measurements were made. Total length (TL): linear distance from anterior margin of pronotum to apex of elytra, pronotal width. (PW): maximum linear transverse distance and elytral width. Elytral width (EW): maximum linear transverse distance. All measurements are given in millimeters. Locality labels are cited in the original version.

The ant species used in the present paper are identified by Dr Mostafa R. Sharaf (Plant Protection Department, College of Food and Agriculture Sciences, King Saud University, Riyadh, Saudi Arabia) and the nomenclature follow the online catalogue (Bolton 2020).

Specimens of the species described were labeled as follows: “HOLOTYPE” [or “PARATYPE,” respectively] *Thorictus ‘species_name*’ sp. nov. Jiří Háva & MS Abdel-Dayem det. 2021.”

Male genitalia were not studied. The differential diagnosis of the aedeagi is often problematical and interspecific variation is currently very poorly defined (John 1963). The two species groups considered here were first established by John in 1963.

### Acronyms of depositories

**JHAC** Jiří Háva, Private Entomological Laboratory and Collection, Únětice u Prahy, Prague-West, Czech Republic;

**KSMA**King Saud University Museum of Arthropods, Plant Protection Department, College of Food and Agriculture Sciences, King Saud University, Riyadh, Saudi Arabia.

## Results

### Family Dermestidae Latreille, 1804

**Subfamily Thorictinae Agassiz, 1846**

**Tribe Thaumaphrastini Anderson, 1949**

**Genus *Thorictodes* Reitter, 1875**

#### Thorictodes heydeni

Taxon classificationAnimaliaColeopteraDermestidae

Reitter, 1875

28EF2FAC-EA8C-503D-AAEE-60482C896BB1

##### Material examined.

Saudi Arabia • 1 ex; Riyadh; Nov. 1989; Habib leg.; on ground; J. Háva det.; JHAC • 1 ex; Riyadh, 5 Oct. 1989; in animal dung; J. Háva det.; KSMA.

##### Remarks.

**Figure 1.**
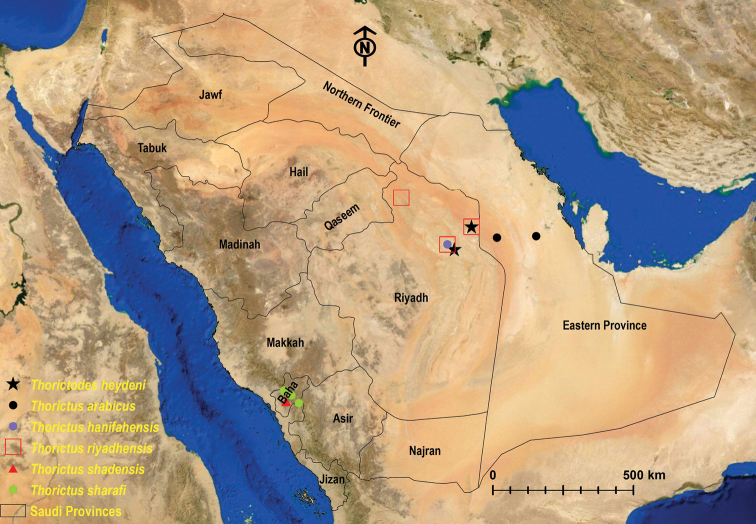
Map of Saudi Arabian provinces and distribution of Thorictinae species.

Cosmopolitan species (Háva 2020a). In Saudi Arabia, this species is only distributed in the Riyadh Province (Fig. 1) and it has been reported at Rawdhat Khorim National Park, Ramah, Riyadh (Abdel-Dayem et al. 2017).

### Tribe Thorictini Agassiz, 1846

**Genus *Thorictus* Germar, 1834**

***castaneus* species group**

#### Thorictus arabicus

Taxon classificationAnimaliaColeopteraDermestidae

Háva, 2010

54C999BE-5A34-57C6-BB25-3AF7C3CD4BD2

Fig. 2

##### Material examined.

Saudi Arabia • 1 ex; Eastern Province, Khuris; [25.08667°N, 48.04306°E]; J. Háva det.; KSMA.

**Figure 2.**
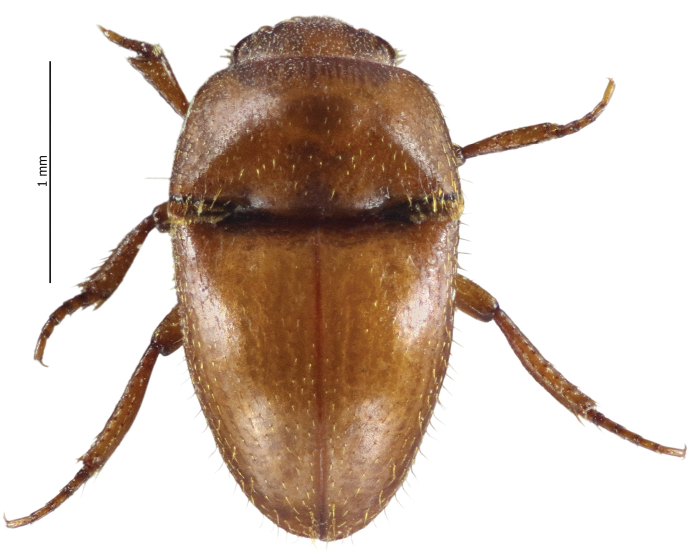
Habitus dorsal aspect of *Thorictus arabicus* Háva, 2010.

##### Remasrks.

An endemic species to Saudi Arabia that was originally described from the Eastern Province (Háva 2010) (Fig. 1). The species was erroneously published by Abdel-Dayem et. al (2017) as *T. castaneus* Germar, 1834, a Mediterranean species that has been reported in Algeria, Egypt, Greece, Morocco, and Syria (Háva 2015a).

#### Thorictus riyadhensis

Taxon classificationAnimaliaColeopteraDermestidae

Háva & Abdel-Dayem sp. nov.

B92425C4-9A4E-53A5-900B-9BBE4F397B67

http://zoobank.org/6018C111-2A2F-43D8-A506-E885A4B9120A

Figs 3 , 4

##### Material examined.

***Holotype.*** Saudi Arabia • 1 ex; Riyadh Province, Wadi Hanifah, WHS04 [location near Waseel]; 24.86682°N, 46.45959°E; alt. 694.942 m; 29 Apr. 2015; Abdel-Dayem M et al. leg.; pitfall trap; KSMA. ***Paratypes.*** Saudi Arabia • 1 ex; Riyadh Province, Az Zulfi, Rawdat Al Sabalh; 26°22.040'N, 44°59.137'E; alt. 670 m; 31 Jan. 2016; Abdel-Dayem M et al. leg.; pitfall trap; KSMA • 1 ex; Riyadh Province, Rhodet Khorim; 25°25.943'N, 47°13.863'E; alt. 572 m; 28 Apr. 2012; pitfall trap, *Calotropis procera*; KSMA • 1 ex; same collection data as preceding; 9 Jun. 2012; pitfall trap, *Acacia gerrardii*; KSMA • 1 ex; same collection data as preceding; 26 May 2012; pitfall trap, *Acacia gerrardii*; JHAC • 1 ex; same collection data as preceding; 26 May 2012; PT, *Lycium shawii*; KSMA • 1 ex; same collection data as preceding; 10 Dec. 2011; pitfall trap; KSMA.

##### Description of holotype.

Body small, brown covered by long, yellow setae on dorsal surfaces and short setae on ventral surfaces. Measurements (mm): TL 2.9, PW 1.9, EW 1.9. Head finely punctate with long yellow setae. Labial palpi entirely brown. Antennae brown, with 11 antennomeres; antennal club compact, with three antennomeres. Pronotum as finely punctate as head and covered by long yellow setation. Lateral margin of pronotum not dentate. Pronotum in posterior part near scutellum without bumps. Ventral posterior pronotal angles with long yellow setation. Scutellum not visible from above. Elytra very finely punctate covered by long yellow setation. Each elytron in anterior part near humeri with one large bump. Epipleuron finely punctate, anterior angles with long yellow setation. Prosternum finely punctate. Mesosternum finely punctate, mesosternal bulge. Metasternum finely punctate. Visible abdominal ventrites very finely punctate and covered by long yellow setae. First abdominal ventrite without anterior, longitudinal striation. Legs brown, covered by yellow setae.

**Figure 3.**
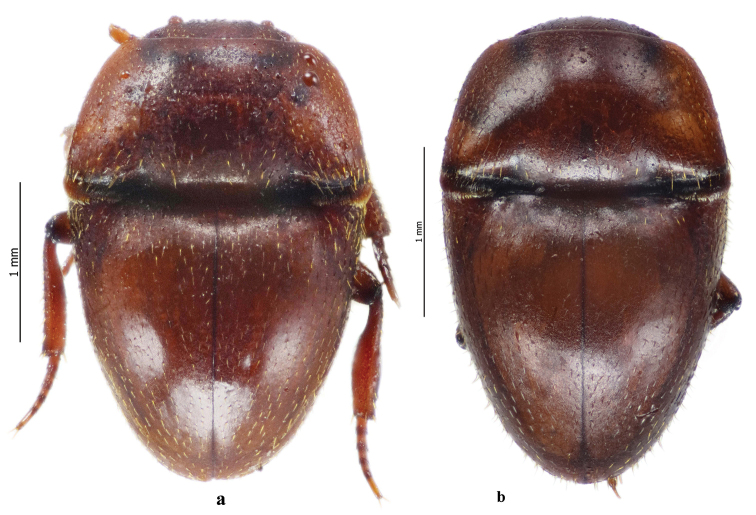
Habitus dorsal aspect of *Thorictus riyadhensis* sp. nov. Háva & Abdel-Dayem **a** specimen from Wadi Hanifah **b** specimen from Rhodet Khorim.

##### Variability.

Body measurements (mm): TL 2.5–2.9, PW 1.5–1.9, EW 1.5–1.9.

##### Differential diagnosis.

The new species belong to the *castaneus* species group, from Saudi Arabia. There is currently only one known species, *Thorictus arabicus* Háva, 2010 but the new species differs from it in the abovementioned characteristics.

**Figure 4.**
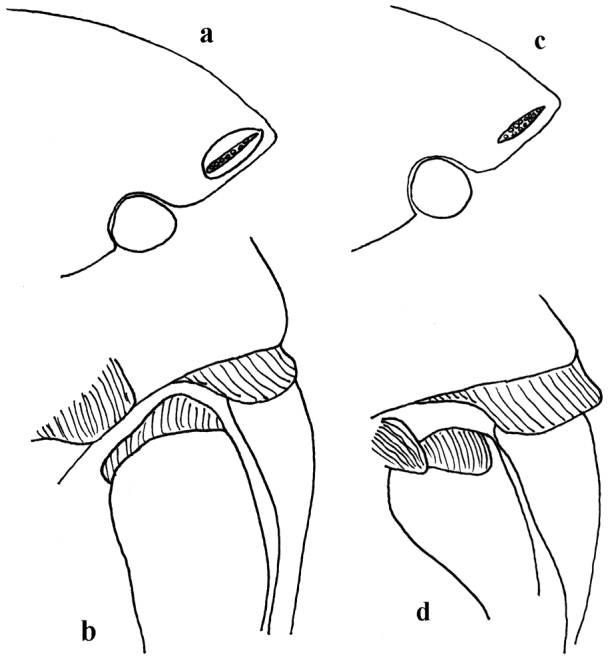
*Thorictus riyadhensis* Háva & Abdel-Dayem, sp. nov. **a** head lateral eye **b** ventral setation on pronotum and metepisternum; *Thorictus arabicus* Háva, 2010 **c** head lateral eye **d** ventral setation on pronotum and metepisternum.

##### Etymology.

Named according to type locality: Riyadh Province.

##### Remarks.

The species was erroneously published by Abdel-Dayem et al. (2017) as *Thorictus foreli* Wasmann, 1894. *Thorictus foreli* is a North African species, occurring in Algeria, Morocco, and Tunisia (Háva 2015a), so it is excluded from the fauna of Saudi Arabia.

##### Ecological notes.

**Figure 5.**
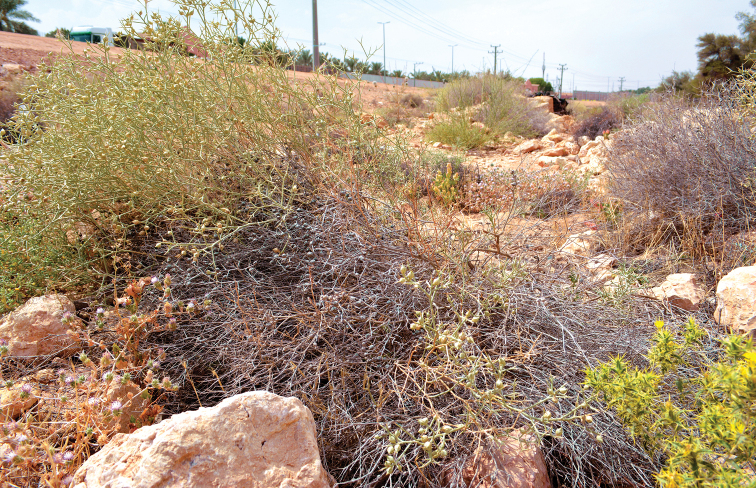
Habitat of *Thorictus riyadhensis* Háva & Abdel-Dayem, sp. nov. holotype in Wadi Hanifah, Ad Diriyah, Riyadh Province, at an elevation of 695 m. *Carthamus oxyacantha* M. Bieb. (Asteraceae) in the bottom right corner, and *Zilla spinosa* (L.) Prantl (Brassicaceae) in the foreground.

**Figure 6.**
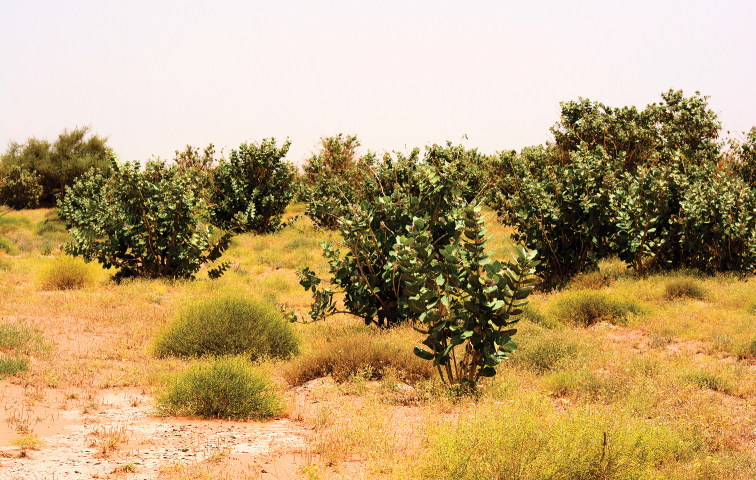
Habitat of *Thorictus riyadhensis* Háva & Abdel-Dayem, sp. nov. paratype in Rawdat Al Sabalh, Az Zulfi, Riyadh Province, at 670 m elevation. *Calotropis procera* (Aiton) W.T. Aiton (Apocynaceae) in background, and *Pulicaria undulata* (L.) C. A. Mey. (Asteraceae) in the foreground and among the shrubs.

**Figure 7.**
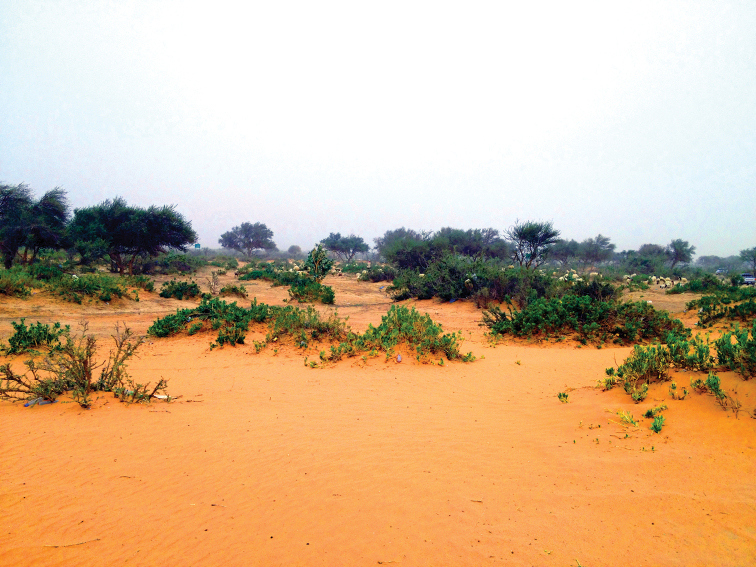
Habitat of *Thorictus riyadhensis* Háva & Abdel-Dayem, sp. nov. paratype in Rhodet Khorim, Ramah, Riyadh Province, at an elevation of 572 m. Trees of *Acacia gerrardii* Benth. (Fabaceae) in the background, shrubs of *Calotropis procera* (Aiton) W. T. Aiton (Apocynaceae) and *Ziziphus nummularia* (Burm. f) Wight & Arn. (Rhamnaceae), shrub of *Lycium shawii* Roem. & Schult. (Solanaceae) in the left corner of the bottom, and *Rhazya stricta* Decne. (Apocynaceae) in the foreground and middle.

The holotype was found in Wadi Hanifah at an area with loam soil covered by *Carthamus oxyacantha* M. Bieb. (Asteraceae) and *Zilla spinosa* (L.) Prantl (Brassicaceae) (Fig. 5). The ant species *Camponotus thoracicus* (Fabricius 1804), *Cataglyphis holgerseni* (Collingwood & Agosti, 1996), *Cataglyphis lividus* (André, 1881), *Lepisiota simplex* (Forel, 1892), and *Monomorium abeillei* (André, 1881) were collected with the holotype in the same pitfall trap. The paratype was found in loam area covered with *Calotropis procera* (Aiton) W.T. Aiton (Apocynaceae) and *Pulicaria undulata* (L.) C. A. Mey. (Asteraceae) at Rawdat Al Sabalh (Fig. 6). While at Rhodet Khorim, the paratypes were collected from a sandy area using pitfall traps under canopies of *Acacia gerrardii* Benth. (Fabaceae), *Calotropis procera* (Aiton) W.T. Aiton, and *Lycium shawii* Roem. & Schult. (Solanaceae) (Fig. 7). Ant species of *Camponotus sericeus* (Fabricius, 1798), *Camponotus xerxes* Forel, 1904, *Cataglyphis fisheri* Sharaf & Aldawood, 2015, *Cataglyphis lividus* (André, 1881), *Cataglyphis niger* (André, 1881), *Cataglyphis viaticoides* (André, 1881), *Lepisiota dammama* Collingwood & Agosti, 1996, *Lepisiota simplex* (Forel, 1892), *Tetramorium chefketi* Forel, 1911, *Tetramorium sericeiventre* Emery, 1877, and *Trichomyrmex mayri* (Forel, 1902) were caught with the paratypes from the same pitfall traps. *Thorictus riyadhensis* was collected during December and January, and from April to June.

##### Geographical distribution.

This new Thorictini species is known from Najd Plateau, Central Saudi Arabia, Riyadh Province (Fig. 1), at Wadi Hanifah (Ad Diriyah), Rawdat Al Sabalh (Az Zulfi), and Rhodet Khorim (Ramah).

### *orientalis* species group

#### Thorictus hanifahensis

Taxon classificationAnimaliaColeopteraDermestidae

Háva & Abdel-Dayem sp. nov.

042022D2-1ADE-5903-BE9E-E293CDF5EEB0

http://zoobank.org/613E430C-72E7-4983-8FD9-A9A4A2F47E54

Figs 8 , 9

##### Material examined.

***Holotype.*** Saudi Arabia • 1 ex; Riyadh Province, Wadi Hanifah, WHS01 [location near Waseel]; 24.87011°N, 46.456775°E; alt. 707.051 m; 12 Oct. 2015; Abdel-Dayem M et al. leg.; pitfall trap; KSMA.

##### Description of holotype.

Body small, brown covered by long yellow setae on dorsal surfaces and short setae on ventral surfaces. Measurements (mm): TL 2.8, PW 1.6, EW 1.6. Head finely punctate with long yellow setae. Labial palpi entirely brown. Antennae brown, with 11 antennomeres, antennal club compact with three antennomeres. Pronotum as finely punctate as head, covered by long yellow setation. Lateral margin of pronotum not dentate. Pronotum in posterior part near scutellum without bumps. Ventral posterior pronotal angles with long yellow setation. Scutellum not visible from above. Elytra very finely punctate covered by long yellow setation. Each elytron in anterior part near humeri with one very small bump. Epipleuron finely punctate, anterior angles with long yellow setation. Prosternum finely punctate. Mesosternum finely punctate, mesosternal bulge. Metasternum finely punctate. Visible abdominal ventrites very finely punctate, covered by long yellow setae. First abdominal ventrite with anterior, longitudinal striation. Legs brown, covered by yellow setae.

**Figure 8.**
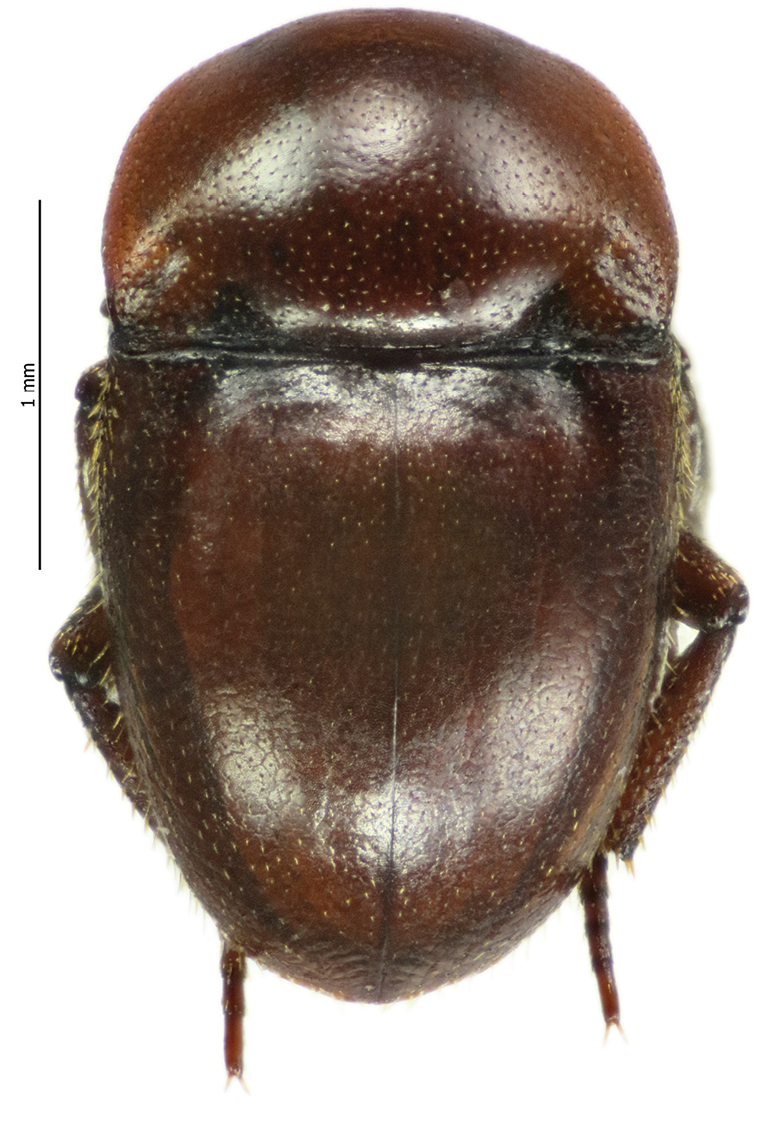
Habitus dorsal aspect of *Thorictus hanifahensis* Háva & Abdel-Dayem, sp. nov.

##### Differential diagnosis.

The new species is similar to *T munganasti* Reitter, 1908 (Egypt) but differs from it by the abovementioned characteristics.

**Figure 9.** *Thorictus hanifahensis* Háva & Abdel-Dayem, sp. nov. **a** head lateral eye **b** ventral setation on pronotum and metepisternum, *T munganasti* (Reitter 1908) **c** head lateral eye (according to John 1963) **d** ventral setation on pronotum and metepisternum (according to John 1963).

##### Etymology.

Named according to type locality: Wadi Hanifah.

##### Ecological notes.

**Figure 10.**
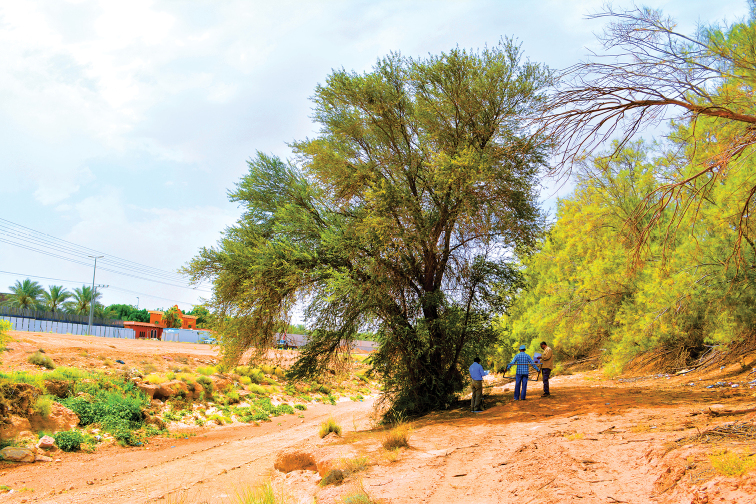
Habitat of *Thorictus hanifahensis* Háva & Abdel-Dayem, sp. nov. holotype in Central Saudi Arabia, Riyadh Province, Wadi Hanifah, at 707 m elevation. *Acacia gerrardii Benth*. (Fabaceae) in the middle foreground and *Tamarix senegalensis* DC. (Tamaricaceae) on the right.

This species was found in an area with loam texture, which is dominated by *Tamarix senegalensis* DC. (Tamaricaceae) and some *Acacia gerrardii* Benth. (Fabaceae) (Fig. 10). The single specimen was collected by pitfall trap in October, along with the ant species *Camponotus sericeus* (Fabricius, 1798), *Camponotus thoracicus* (Fabricius, 1804), *Cataglyphis holgerseni* Collingwood & Agosti, 1996, *Cataglyphis livida* (André, 1881), *Lepisiota simplex* (Forel, 1892), *Monomorium abeillei* André, 1881, *Monomorium niloticum* Emery, 1881, *Tetramorium caespitum* (Linnaeus, 1758), and *Trichomyrmex mayri* (Forel, 1902) in the same trap.

##### Geographical distribution.

*Thorictus hanifahensis* Háva & Abdel-Dayem, sp. nov. is known only from its type locality in central Saudi Arabia, at Wadi Hanifah in the Ad Diriyah Governorate, Riyadh Province (Fig. 1).

#### Thorictus peyerimhoffi

Taxon classificationAnimaliaColeopteraDermestidae

Chobaut, 1904

F1EB7144-355C-5A7B-ADBC-A6D7F5B2EDAC

##### Remarks.

Chobaut (1904) described the species from “Kasr-er-Rabbat in Arabia”, a locality located in Jordan (Tronquet 1998). The species is excluded from the fauna of Saudi Arabia.

#### Thorictus shadensis

Taxon classificationAnimaliaColeopteraDermestidae

Háva & Abdel-Dayem sp. nov.

4A351517-5B4E-533A-B88D-40962DA2773C

http://zoobank.org/B2CC6ACC-DA67-49F7-A14D-4BA09F608F0B

Figs 11 , 12

##### Material examined.

***Holotype***. Saudi Arabia • 1 ex; Al Bahah, Shada Al Ala Nature Reserve; 19°50.710'N, 41°18.267'E; alt. 1474 m; 5 Jul. 2014; Al Dhafer H, Fadl H, Abadel-Dayem M, El Torkey A, El Gharbawy A leg.; pitfall trap; KSMA. ***Paratypes***. Saudi Arabia • 1 ex; Al Bahah, Shada Al Ala Nature Reserve; 19°50.329'N, 41°18.604'E; alt. 1563 m; 23 Apr. 2014; Al Dhafer H, Fadl H, Abad Eidayem M, El Torkey A, El Gharbawy A leg.; pitfall trap; KSMA • 1 ex; same collection data as preceding; 19°51.762'N, 41°18.089'E; alt. 1225 m; 23 Apr. 2014; Al Dhafer H, Fadl H, Abad Eidayem M, El Torkey A, El Gharbawy A leg.; pitfall trap; JHAC.

**Figure 11.**
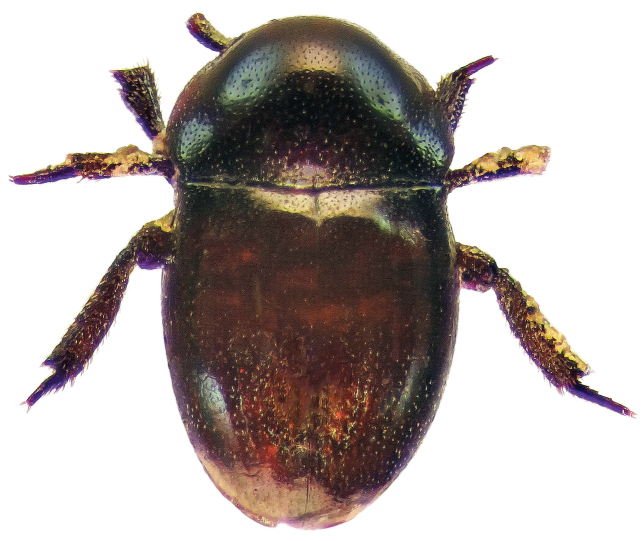
Habitus dorsal aspect of *Thorictus shadensis* Háva & Abdel-Dayem, sp. nov.

##### Description of holotype.

Body small, brown covered by short yellow setae on dorsal surfaces and short setae on ventral surfaces. Measurements (mm): TL 2.1, PW 1.1, EW 1.2. Head finely punctate with short yellow setae. Labial palpi entirely brown. Antennae brown, with 11 antennomeres, antennal club compact with three antennomeres. Pronotum as finely punctate as head, covered by short yellow setation. Lateral margin of pronotum not dentate. Pronotum in posterior part near scutellum without bumps. Ventral posterior pronotal angles without long yellow setation. Scutellum not visible from above. Elytra very finely punctate covered by short yellow setation. Each elytron in anterior part near humeri with one very small bump. Epipleuron finely punctate, anterior angles with short yellow setation and small bump. Prosternum finely punctate. Mesosternum finely punctate, mesosternal bulge. Metasternum finely punctate. Visible abdominal ventrites very finely punctate, covered by short yellow setae. First abdominal ventrite with anterior, longitudinal striation. Legs brown, covered by yellow setae.

**Figure 12.**
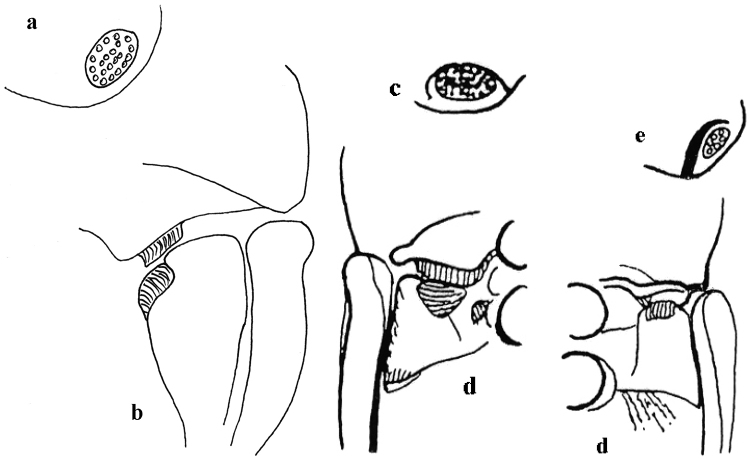
*Thorictus shadensis* Háva & Abdel-Dayem, sp. nov. **a** head lateral eye **b** ventral setation on pronotum and metepisternum, *T abyssinicus* John, 1963 **c** head lateral eye **d** ventral setation on pronotum and metepisternum, *T dilatipennis* Reitter, 1881 **e** head lateral eye (according to John 1963) **f** ventral setation on pronotum and metepisternum (according to John 1963).

##### Variability.

Body measurements (mm): TL 2.1–2.2, PW 1.0–1.1, EW 1.1–1.2.

##### Differential diagnosis.

The new species belongs to the *orientalis* species group and is very similar to two other species: *T abyssinicus* John, 1963 (Ethiopia) and *T dilatipennis* Reitter, 1881 (Egypt, Syria), but differs from them by the abovementioned characteristics.

##### Etymology.

Named according to type locality: Shada Al Ala Nature Reserve.

##### Ecological notes.

**Figure 13.**
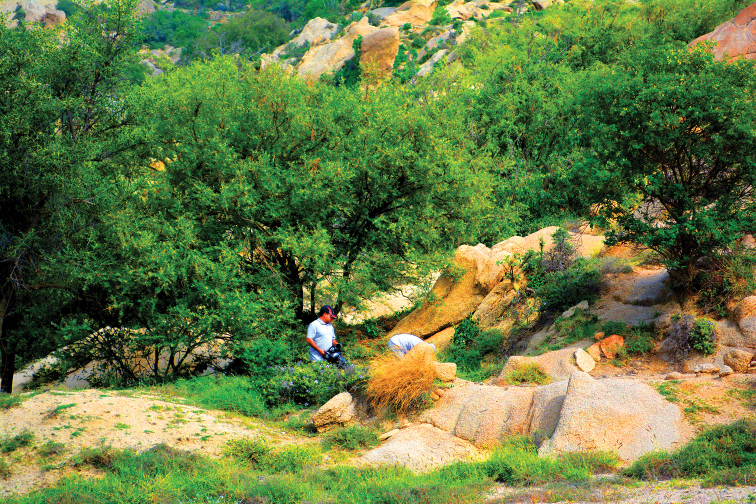
*Acacia* thorn woodlands, the type locality of *Thorictus shadensis* Háva & Abdel-Dayem, sp. nov. holotype at Shada Al Ala Nature Reserve on the Shada Mountain, Baha Province, southwestern Saudi Arabia, at an elevation of 695 m.

**Figure 14.**
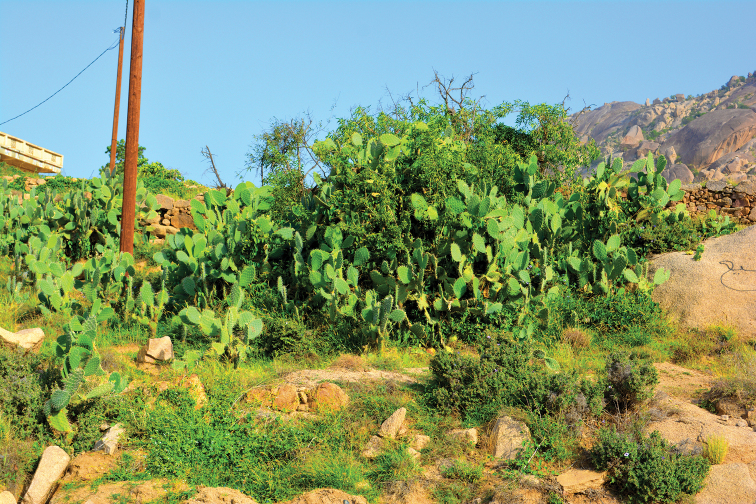
Barbary fig or cactus pear shrubs, *Opuntia ficus-indica* (L.) Mill., the type locality of paratype of *Thorictus shadensis* Háva & Abdel-Dayem, sp. nov. at Shada Al Ala Nature Reserve on the Shada Mountain, Baha Province, southwestern Saudi Arabia, at 1563 m elevation.

The adult beetles were found during April and July at elevations of 1225–1563 m in the Shada Al Ala Nature Reserve. The specimens were collected by pitfall traps in steep slopes covered with vegetation dominated by *Acacia* thorn woodlands and shrubs of Barbary fig or cactus pear, *Opuntia ficus-indica* (L.) Mill. (Cactaceae) (Figs 13, 14). This new species was caught with ant species of *Camponotus aegyptiacus* (Emery, 1915), *Crematogaster* sp., *Lepisiota obtusa* (Emery, 1901), *Monomorium jizane* Collingwood & Agosti, 1996, *Monomorium rabirium* Bolton, 1987, *Pheidole* sp., and *Tetramorium simillium* (Smith, 1851) in the same pitfall traps.

##### Geographical distribution.

This species is only known from the type locality in the Shada Al Ala Nature Reserve, on the Shada Mountain, in the west of the Sarawat Mountains at Al Mekhwah, Baha Province (Fig. 1).

#### Thorictus sharafi

Taxon classificationAnimaliaColeopteraDermestidae

Háva & Abdel-Dayem sp. nov.

FD63B466-80D7-5E57-9728-BD979D857994

http://zoobank.org/75B52C5E-77B0-4BE7-9074-48B32BFE779C

Figs 15 , 16

##### Material examined.

***Holotype*.** Saudi Arabia • 1 ex; Baha Region, Alqamh Park, Belgershi; 19°48.407'N, 41°42.718'E; alt. 1931 m; 17 May 2010; Dr M.R. Sharaf leg.; KSMA. ***Paratypes*.** Saudi Arabia • 1 ex; Baha Region, Amadan, Mandaq; 20°12.163'N, 41°13.906'E; alt. 1881 m; 19 May 2010; Dr M.R. Sharaf leg.; KSMA • 1 ex; Baha Region, Shohba forest; 20°02.723'N, 41°28.565'E; alt. 2324 m; 14 May 2010; Dr M.R. Sharaf leg. JHAC.

##### Description of holotype.

Body small, brown covered by short yellow setae on dorsal surfaces and short setae on ventral surfaces. Measurements (mm): TL 2.1, PW 1.1, EW 1.2. Head finely punctate with short yellow setae. Labial palpi entirely brown. Antennae brown, with 11 antennomeres, antennal club compact with three antennomeres. Pronotum as finely punctate as head, covered by short yellow setation. Lateral margin of pronotum not dentate. Pronotum in posterior part near scutellum without bumps. Ventral posterior pronotal angles without long yellow setation. Scutellum not visible from above. Elytra very finely punctate covered by short yellow setation. Each elytron in anterior part near humeri with one very small bump. Epipleuron finely punctate; anterior angles with short yellow setation. Prosternum finely punctate. Mesosternum finely punctate, mesosternal bulge. Metasternum finely punctate. Visible abdominal ventrites very finely punctate, covered by short yellow setae. First abdominal ventrite with anterior, longitudinal striation. Legs brown, covered by yellow setae.

**Figure 15.**
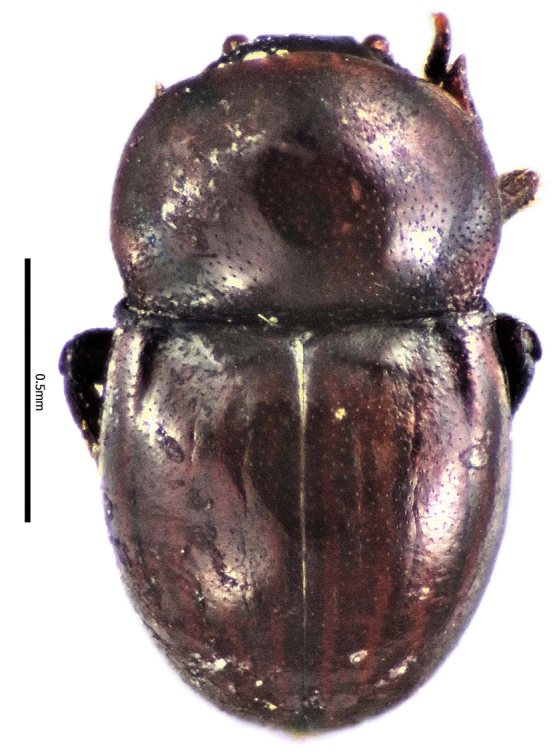
Habitus dorsal aspect of *Thorictus sharafi* Háva & Abdel-Dayem, sp. nov.

##### Variability.

Body measurements (mm): TL 2.1–2.2, PW 1.0–1.1, EW 1.1–1.2.

**Figures 16.**
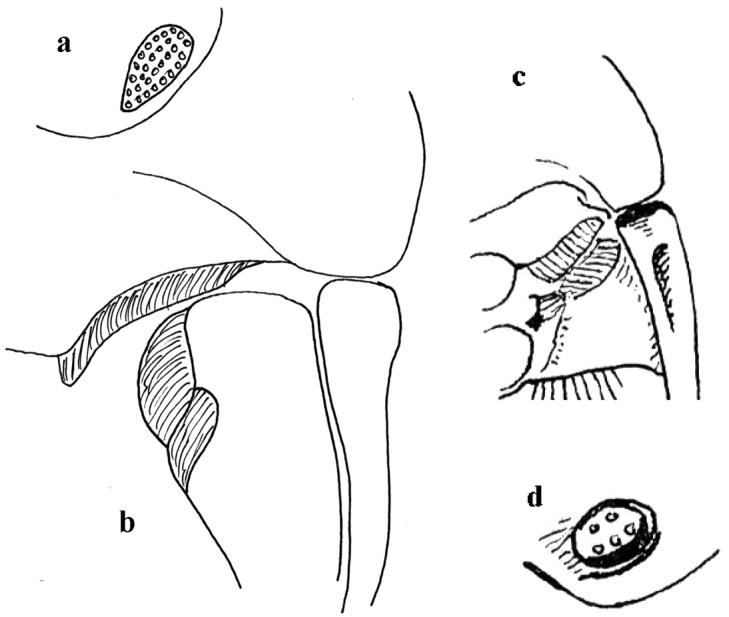
*Thorictus sharafi* Háva & Abdel-Dayem, sp. nov. **a** head lateral eye **b** ventral setation on pronotum and metepisternum, *T dohrni* John, 1965 **c** head lateral eye **d** ventral setation on pronotum and metepisternum (according to John 1963).

##### Differential diagnosis.

This new species is similar to *T dohrni* John, 1965 (Ethiopia), but differs from it by the abovementioned characteristics.

##### Etymology.

The specific epithet is a Latinized noun in the genitive case in the masculine form based on the honorific name “Dr Mostafa Sharaf,” who collected the specimens of this species.

##### Ecological notes.

**Figure 17.**
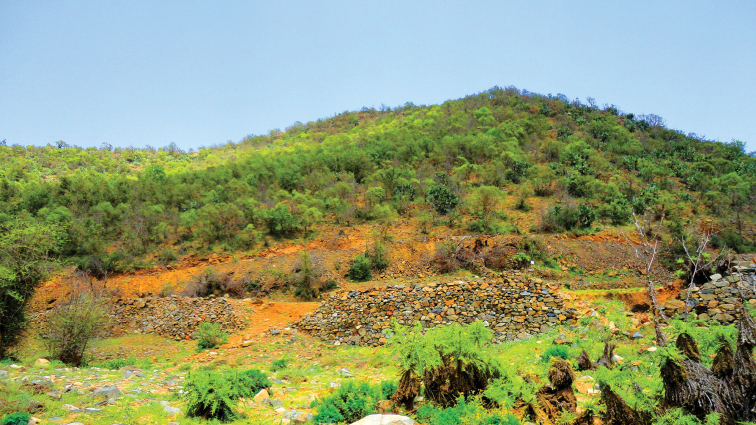
Photo of African pencil cedar forest, *Juniperus procera* Hochst. ex Endl. (Cupressaceae), living habitat of *Thorictus sharafi* Háva & Abdel-Dayem, sp. nov. holotype at Alqamh Park, Belgershi, Baha Province, in the mountains of southwestern Saudi Arabia, at 1931 m elevation.

**Figure 18.**
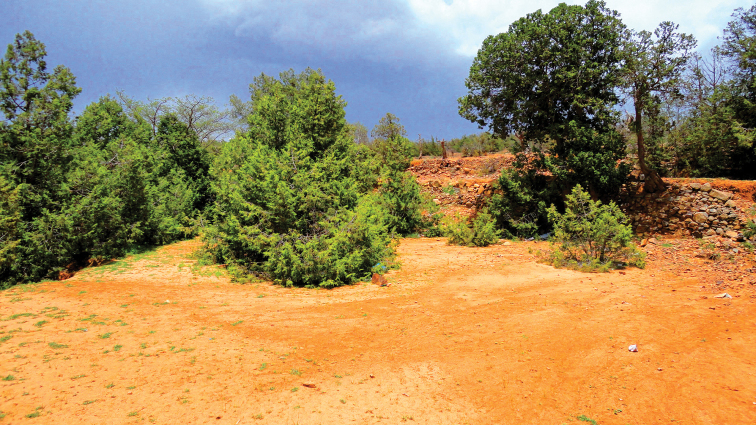
Living habitat of *Thorictus sharafi* Háva & Abdel-Dayem sp. nov. paratype in the mountains of southwestern Saudi Arabia, Baha Province, within the African pencil cedar forest, *Juniperus procera* Hochst. ex Endl. (Cupressaceae) of Shohba forest at 2324 m elevation.

This new species inhabits areas at elevations of 1881–2324 m within the African pencil cedar forest, *Juniperus procera* Hochst. ex Endl. (Cupressaceae) in the Baha Province (Figs 17, 18). All specimens were collected by hand-picking under stones during May. Host unknown.

##### Geographical distribution.

This species is known only from its type locality in the mountains of southwestern Saudi Arabia, at Alqamh Park, Amadan, and Shohba Forest in the Baha Province (Fig. 1).

## Discussion

The study of the insect fauna of Saudi Arabia began with Buttiker and Wittmer (1979) who surveyed insects throughout the country. These surveys leading the entomological exploration of Saudi Arabia carried on for well over a half century thereafter. However, the Thorictinae fauna remains insufficiently known due to a lack of adequate dedicated survey. Based on the examination of the literature records (Háva 2010, 2021; Abdel-Dayem et al. 2017) and collected specimens, the total number of Thorictinae species known from Saudi Arabia now stands at six species (Table 1). These species belong to two genera *Thorictodes* Reitter, 1875 and *Thorictus* Germar, 1834 under two tribes Thaumaphrastini and Thorictini, respectively (Háva 2020a). Of these species, four are new to science, namely *T. hanifahensis* Háva & Abdel-Dayem, sp. nov., *T. riyadhensis* Háva & Abdel-Dayem, sp. nov., *T. shadensis* Háva & Abdel-Dayem, sp. nov., and *T. sharafi* Háva & Abdel-Dayem, sp. nov. A cosmopolitan species *Thorictodes heydeni* Reitter, 1875 is known and only *T. arabicus* Háva, 2010 is endemic to the Saudi fauna. Herein we exclude three species from the fauna of Saudi Arabia, either due to erroneous publication such as *T. castaneus* Germar, 1834 and *T. foreli* Wasmann, 1894 (Abdel-Dayem et. al 2017) or taxonomic misplacement such as *Thorictus peyerimhoffi* Chobaut, 1904 (Háva 2010). Although the Arabian Peninsula occupies almost twice the area of Iran, the number of species recorded from the Arabian Peninsula (7 species Háva 2020a) is less than that recorded from Iran (10 species; Háva and Švarc 2020). No common species are shared between the two regions.

**Table 1.** List of Thorictinae species from the Arabian Peninsula. Indication: recorded (*), not recorded (–), excluded (X).

	Kuwait	Saudi Arabia	Yemen		Oman	United Arab Emirates	Qatar
			Continental Yemen	Soqotra Island			
**Subfamily Thorictinae**							
**Tribe Thorictini**							
*Thorictus arabicus* Háva, 2010	–	*	–	–	–	–	–
*Thorictus hanifahensis* Háva & Abdel-Dayem, sp. nov.	–	*	–	–	–	–	–
*Thorictus omanensis* Háva, 2021	–	–	–	–	*	–	–
*Thorictus riyadhensis* Háva & Abdel-Dayem, sp. nov.	–	*	–	–	–	–	–
*Thorictus shadensis* Háva & Abdel-Dayem, sp. nov.	–	*	–	–	–	–	–
*Thorictus sharafi* Háva & Abdel-Dayem, sp. nov.	–	*	–	–	–	–	–
**Tribe Thaumaphrastini**							
*Thorictodes heydeni* Reitter, 1875	–	*	–	–	–	–	–
**Excluded species**							
*Thorictus castaneus* Germar, 1834	–	X	–	–	–	–	–
*Thorictus foreli* Wasmann, 1894	–	X	–	–	–	–	–
*Thorictus peyerimhoffi* Chobaut, 1904	–	X	–	–	–	–	–

The male genitalia are very important for differential diagnoses in insect taxonomy. But interspecific variation in male genitalia within species of Thorictinae leads to problems in the differential diagnosis of the aedeagi (John 1963). Thus, we did not investigate the male genitalia of the studied specimens in this work.

We reported the ant species captured with the beetle species from the same pitfall trap. The host species of ants were not recognized during this study due to the fact that these specimens were collected accidentally from field surveys. The *Thoricuts* species are phoretic and obligate myrmecophiles, either generalists or specialists (Lenoir et al. 2013) and they are considered detritivorous (Sánchez-Piñero and Gómez 1995). The members of *Thorictus* are generally associated with various *Cataglyphis* species (Lenoir et al. 2013).

## Supplementary Material

XML Treatment for Thorictodes heydeni

XML Treatment for Thorictus arabicus

XML Treatment for Thorictus riyadhensis

XML Treatment for Thorictus hanifahensis

XML Treatment for Thorictus peyerimhoffi

XML Treatment for Thorictus shadensis

XML Treatment for Thorictus sharafi
